# Natural history of type 1 diabetes on an immunodysregulatory background with genetic alteration in B-cell activating factor receptor: A case report

**DOI:** 10.3389/fimmu.2022.952715

**Published:** 2022-08-26

**Authors:** Biagio Di Lorenzo, Lucia Pacillo, Giulia Milardi, Tatiana Jofra, Silvia Di Cesare, Jolanda Gerosa, Ilaria Marzinotto, Ettore Zapparoli, Beatrice Rivalta, Cristina Cifaldi, Federica Barzaghi, Carmela Giancotta, Paola Zangari, Novella Rapini, Annalisa Deodati, Giada Amodio, Laura Passerini, Paola Carrera, Silvia Gregori, Paolo Palma, Andrea Finocchi, Vito Lampasona, Maria Pia Cicalese, Riccardo Schiaffini, Gigliola Di Matteo, Ivan Merelli, Matteo Barcella, Alessandro Aiuti, Lorenzo Piemonti, Caterina Cancrini, Georgia Fousteri

**Affiliations:** ^1^ Diabetes Research Institute, Istituto di Ricovero e Cura a Carattere Scientifico Ospedale San Raffaele, Milan, Italy; ^2^ Department of Systems Medicine, University of Rome Tor Vergata, Rome, Italy; ^3^ Academic Department of Pediatrics (DPUO), Research Unit of Clinical Immunology and Vaccinology, Bambino Gesú Children’s Hospital, Istituto di Ricovero e Cura a Carattere Scientifico, Rome, Italy; ^4^ Center for Omics Sciences, Istituto di Ricovero e Cura a Carattere Scientifico Ospedale San Raffaele, Milano, Italy; ^5^ San Raffaele Telethon Institute for Gene Therapy (SR-Tiget), Istituto di Ricovero e Cura a Carattere Scientifico San Raffaele Scientific Institute, Milan, Italy; ^6^ Pediatric Immunohematology and Bone Marrow Transplantation Unit, Istituto di Ricovero e Cura a Carattere Scientifico San Raffaele Scientific Institute, Milan, Italy; ^7^ Unit of Endocrinology, Bambino Gesù Children’s Hospital, Istituto di Ricovero e Cura a Carattere Scientifico, Rome, Italy; ^8^ Unit of Genomics for Human Disease Diagnosis and Laboratory of Clinical Molecular Biology, Istituto di Ricovero e Cura a Carattere Scientifico Ospedale San Raffaele, Milan, Italy; ^9^ Faculty of Medicine, University Vita-Salute San Raffaele, Milan, Italy; ^10^ Department of Bioinformatics, Institute for Biomedical Technologies National Research Council, Segrate, Italy

**Keywords:** type 1 diabetes (T1D), common variable immunodeficiency (CVID), BAFFR mutation, islet autoimmunity, circulating T follicular helper cells (cTfh)

## Abstract

The immunological events leading to type 1 diabetes (T1D) are complex and heterogeneous, underscoring the necessity to study rare cases to improve our understanding. Here, we report the case of a 16-year-old patient who showed glycosuria during a regular checkup. Upon further evaluation, stage 2 T1D, autoimmune thrombocytopenic purpura (AITP), and common variable immunodeficiency (CVID) were diagnosed. The patient underwent low carb diet, losing > 8 kg, and was placed on Ig replacement therapy. Anti-CD20 monoclonal antibody (Rituximab, RTX) was administered 2 years after diagnosis to treat peripheral polyneuropathy, whereas an atypical mycobacteriosis manifested 4 years after diagnosis and was managed with prolonged antibiotic treatment. In the fifth year of monitoring, the patient progressed to insulin dependency despite ZnT8A autoantibody resolution and IA-2A and GADA autoantibody decline. The patient had low T1D genetic risk score (GRS = 0.22817) and absence of human leukocyte antigen (HLA) DR3/DR4-DQ8. Genetic analysis identified the monoallelic mutation H159Y in *TNFRSF13C*, a gene encoding B-cell activating factor receptor (BAFFR). Significant reduced blood B-cell numbers and BAFFR levels were observed in line with a dysregulation in BAFF–BAFFR signaling. The elevated frequency of PD-1^+^ dysfunctional Tfh cells composed predominantly by Th1 phenotype was observed at disease onset and during follow-up. This case report describes a patient progressing to T1D on a BAFFR-mediated immunodysregulatory background, suggesting a role of BAFF–BAFFR signaling in islet-specific tolerance and T1D progression.

## Introduction

Type 1 diabetes (T1D) is a disease of multifactorial origin caused by the autoimmune destruction of insulin-producing pancreatic β cells. Several immune players have been identified as contributors to the disease immunopathogenesis, involving both the innate and adaptive arms of the immune system (1–3). T cells seem to play a dominant role during the disease pathogenesis and are directly involved in the pancreatic β-cell killing. The possible role of B cells and autoantibodies (AAbs) in T1D remains elusive, which are thought to act mainly as antigen-presenting cells. Islet-specific AAbs—such as glutamic acid decarboxylase 65 (GAD65), insulin, the tyrosine phosphatase–like autoantigen IA-2, or the ZnT8—are the most reliable biomarkers for disease diagnosis and prediction (4, 5). Today, T1D patients can be subdivided into three stages based on the presence of islet-specific AAbs and impaired glucose tolerance: stage 1 T1D, with individuals positive for at least two islet-specific AAbs and no metabolic dysregulation; stage 2 T1D, with individuals who developed impaired glucose tolerance; and stage 3 T1D, with individuals with multiple AAb-positive and fasting hyperglycemia (clinical diabetes) (6, 7)

A poorly defined interaction between genetic and environmental factors underlies T1D pathogenesis. HLA accounts for the majority of T1D genetic risk, whereas single-nucleotide polymorphisms (SNPs) in non-HLA genes, such as *INS*, *PTPN22*, *IL2RA*, *IFIH1*, and *CTLA4*, are considered additional contributing genetic factors (8, 9). Recently, several T1D genetic risk scores (GRSs) have been developed based on HLA and non-HLA T1D-risk genes (30-97 SNPs). These scores can discriminate T1D from type 2 diabetes (T2D), monogenic diabetes from T1D, and monogenic autoimmunity from early onset T1D associated with poly-autoimmunity (10, 11).

Common variable immunodeficiency (CVID) is a heterogenous disease classified as predominantly antibody deficiency (12), with a broad variety of clinical spectrum, characterized by low levels of immunoglobulins (Ig) and failure to produce antigen-specific antibodies with a normal or low levels of B cells and different involvement of cellular immunity. Reduced B-cell counts, isotype-switched B cells (13, 14) and plasmablasts (15) have been described in individuals affected by CVID. In addition, several T-cell defects have been described that often account for the failed B-cell helper support occurring in germinal centers (GCs) (16–19). Patients with CVID often present autoimmune manifestations, mainly autoimmune cytopenia and inflammatory bowel disease (20). T1D in CVID has been described in a handful of reports, but the underlying mechanism and genetic causes remain unknown (21). In this study, we report a patient who at 16 years of age was diagnosed with stage 2 T1D and CVID. Genetic analyses identified a monoallelic mutation in the B-cell activating factor receptor (BAFFR). T1D GRS analysis showed a reduced risk for T1D, suggesting that the identified BAFFR mutation together with other factors, genetic, and environmental determined the progression to T1D.

## Case description

A healthy 16-year-old man with a Caucasian ethnic background underwent a medical visit for a pre-participation sport evaluation. As part of the checkup, urinalysis was performed, resulting positive for glycosuria (99 mg/dl) but negative for ketones. Biochemical analysis revealed the presence of prediabetes (FPG 120 mg/dl, HbA1c 42 mmol/mol) associated with mild thrombocytopenia (89,000/µl) and microcytemia (MCV 78 fl) that was treated with iron supplementation for 1.5 months. Of note, glycosuria (252 mg/dl), not further addressed, and a platelet count at the lower limit of normal (166,000/µl) were present at the age of 12 years, according to his medical records. Stage 2 T1D was diagnosed by the presence of three islet AAbs (IA-2, GADA, and ZnT8A), dysglycemia (FPG 101 mg/dl, HbA1c 40 mmol/mol), glucose intolerance (FPG 309 mg/dl at 2-h 75-g Oral Glucose Tolerance Test (OGTT)), and a partially impaired insulin secretion (fasting insulin and C-peptide: 15.45 mU/L and 1.85 ng/ml; 2-h 75-g Oral Glucose Tolerance Test insulin and C-peptide: 47.52 mU/L and 3.46 ng/ml). Family history included autoimmune Hashimoto’s thyroiditis (treated with levothyroxine) (father), anti-thyroid peroxidase antibodies (younger brother), and T2DM (maternal grandmother). No signs of celiac disease, atrophic gastritis, or autoimmune thyroid disease were found in the patient. A low-carb diet was recommended with a consequent decline in weight (> 8 kg in a 3-month period) and blood glucose normalization. Concomitant to stage 2 T1D, immune thrombocytopenia (ITP) (PLT 47,000/µl, anti-PLT antibodies positive), and hypogammaglobulinemia (IgG: 323 mg/dl; IgM: 21 mg/dl; IgA: 48 mg/dl) were diagnosed (22). Bone marrow biopsy excluded any lymphoproliferative diseases confirming the ITP diagnosis. Microbiological analysis and EBV serology were negative, except for low copies of HHV6 and Parvovirus B19 in the bone marrow. Two months later, the patient was hospitalized for severe immune thrombocytopenia (platelets: 20,000/µl), which was treated with high-dose intravenous immunoglobulin (IVIg) with a good response. During hospitalization, hypogammaglobulinemia was confirmed (IgG: 344 mg/dl; IgM: 33.10 mg/dl; IgA: 6.92 mg/dl). Immunological investigations showed mild lymphopenia with an increase in memory T-cell subsets and alteration in B-cell maturation, with low memory B-cell frequencies, absent switched memory B cells, and low/absent antigen-specific T-cell responses. In the same year, the patient had experienced recurrent tonsillitis, but his past medical history was negative for severe or recurrent infections, with the exception of laryngospasm episodes in pre-scholar age. Consequently, after excluding other secondary causes and considering the persistence of hypogammaglobulinemia, a clinical diagnosis of CVID was made and he started IVIg replacement therapy.

During a 5-year follow-up, he did not experience any ITP relapses and his platelet count remained stable between 100,000 and 150,000/µl.

Two years after CVID and T1D stage 2 diagnosis, the patient was admitted to the hospital for asymmetric axonal sensitive polyneuropathy, probably triggered by CMV infection, which was managed with high-dose IVIg, RTX, Pregabalin, and Duloxetin. Steroids were not considered due to his comorbidities (pre-clinical diabetes and hypertension). Neurological improvement occurred with a mild persistence of sensitive alterations.

Two years later, an atypical mycobacterial pulmonary infection associated with generalized lymphadenopathy and worsening splenomegaly was discovered and treated with long-time pluri-antibiotic therapy.

The patient remained insulin free for 4 years after the initial prediabetes diagnosis when the dysglycemia evolved into stage 3 T1D (at 21 years of age) marking the start of insulin therapy.

Despite receiving three doses of the anti–SARS-CoV-2 vaccine (the last dose in December 2021) and showing a good humoral and cellular response (23), the patient was infected by SARS-CoV-2 virus in April 2022 and experienced a paucisymptomatic clinical course without the necessity of additional therapies and viral clearance in 15 days. Currently, the patient is on subcutaneous Ig replacement therapy (20 gr/28 days) and insulin Glargine 20 UI/day.

### Timeline

**Figure 1:**
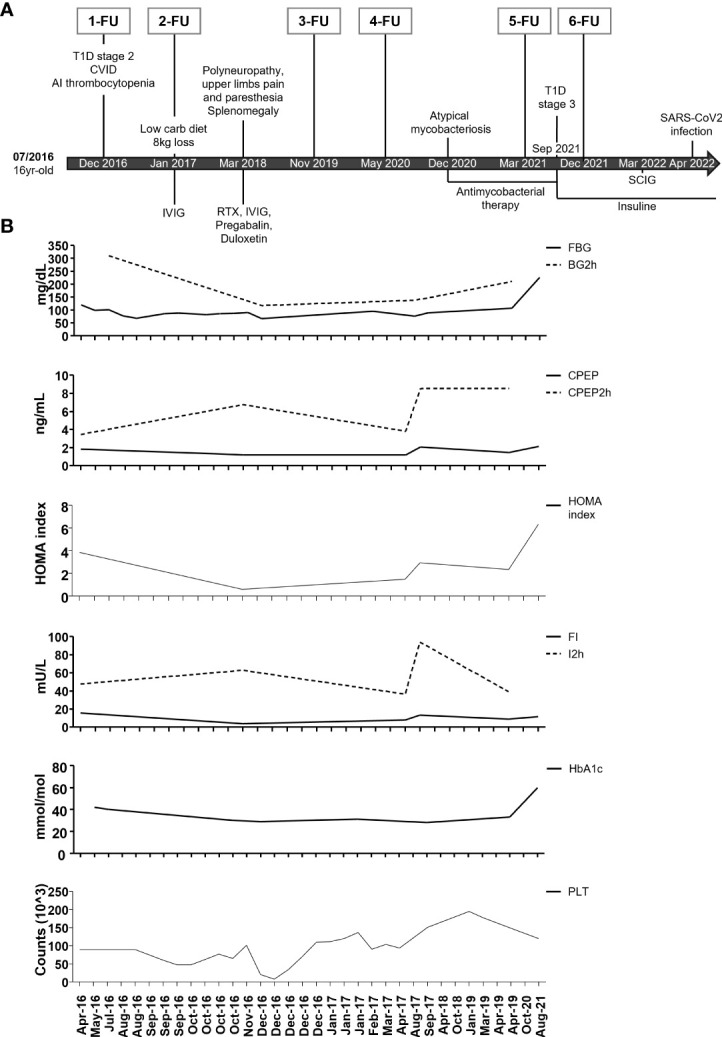
Timeline of clinical events, therapeutic interventions, and diagnostic procedures. **(A)** Summary of major clinical manifestations and therapeutic interventions. IVIG: intravenous immunoglobulins; SCIG: subcutaneous immunoglobulins; RTX: Rituximab. At each follow-up, an extensive immune cell phenotyping was conducted. **(B)** Timeline of fasting blood glucose (FBG), 2-h blood glucose (BG2h), fasting C-peptide (CPEP), and 2-h C-peptide (CPEP2h), HOMA index, fasting insulin (FI) and 2-h insulin (I2h), glycohemoglobin (HbA1c), and platelet count (PLT) from April 2016 to August 2021.

The complete timeline from the time of diagnosis (07/2016) to now is shown in **Figure 1**.

### Genetic assessment

**Figure 2:**
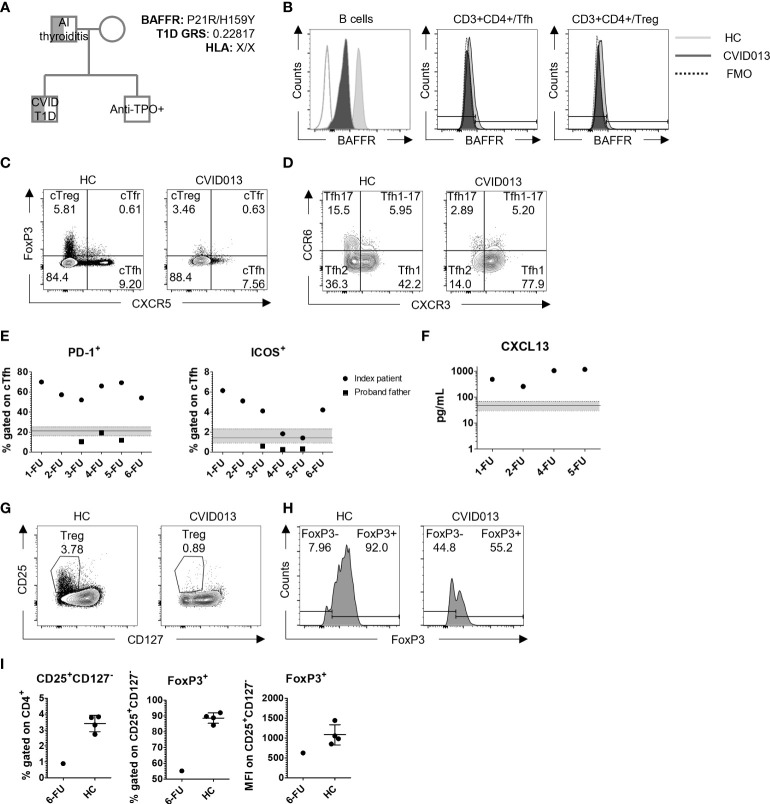
Genetic and immunological characteristics of a patient with CVID and stage 2 T1D. **(A)** Genetic testing identified low T1D GRS (0.22817, HLA: X/X), and H159Y mutation in *BAFFR* inherited in a patrilineal fashion. The father was diagnosed with autoimmune thyroiditis, and the brother was positive for anti-TPO autoantibody production. **(B)** Representative gating strategy to evaluate BAFFR distribution on B cells, Tfh, and T_reg_. White, dark, and light gray slopes for control, HC, and CVID013, respectively. **(C)** FoxP3 and CXCR5 staining on CD3^+^CD4^+^ lymphocytes identifies Tfh (CXCR5^+^FoxP3^-^), Tfr (CXCR5^+^FoxP3^+^), and T_reg_ (CXCR5^-^FoxP3^+^) cells, and **(D)** CXCR3 and CCR6 staining on CD4^+^CXCR5^+^CD45RA^-^. The following subsets were identified: cTfh1 (CXCR3^+^CCR6^-^), cTfh2 (CXCR3^-^CCR6^-^), and cTfh17 (CXCR3^-^CCR6^+^). **(E)** PD-1^+^ and ICOS^+^ expressing cells among cTfh are increased over time compared to the HC group (PD-1^+^ median, IQR = 21.3%, 16.4–25.3, *n =* 65; ICOS^+^ median, IQR = 1.45, 0.91–2.32, *n =* 65). Solid dots and squares represent the index patient and the proband father, respectively. **(F)** CXCL13 was evaluated in plasma by ELISA assay. Stable higher levels of this chemoattractant were detected over time in CVID patient when compared with the HC (median, IQR = 47.68 pg/ml, 29.52–68.24; *n* = 65), represented by the continuous line within the light gray area. (**G, H**) T_reg_ gating strategy based on CD25 and CD127 expression, and FoxP3 expressing cells among CD25^+^CD127^-^ T_reg_. **(I)** CD25^+^CD127^-^ T_reg_ were reduced in the index patient at the first follow-up (HC mean ± *SD* = 3.42% ± 0.51) and expressed lower levels of FoxP3 (mean HC FoxP3 ± *SD* = 88.7% ± 3.3; mean HC FoxP3 MFI ± *SD* = 1080 ± 254.2).

The index patient underwent genetic screening by whole exome sequencing (WES). A monoallelic mutation in *BAFFR* (H159Y) was identified and confirmed by Sanger sequencing. The mother carried the wild-type allele, whereas the father carried the same mutation. Additionally, T1D GRS was calculated by typing 30 common HLA and non-HLA genetic variants associated with T1D, as previously described (10). The index patient did not have a T1D-risk HLA (X/X for DR3/DR4-DQ8) and his T1D GRS score was 0.22817 (**Figure 2A**). Moreover, the monoallelic mutation in BAFFR was associated with reduced gMFI BAFFR expression on the B cell, Tfh, and T regulatory cell (T_reg_) surface as compared with HC. BAFFR decrease was more pronounced in B cells (MFI reduction 82.1%) than in T cells (reduction 15.4%, 18.9%, and 18.5% in Tfh, T_reg_, and Tfr, respectively) (**Figure 2B**). Similar to the index patient, the father expressed reduced levels of BAFFR on the surface of his circulating B cells (**Figure S1**).

### Immunological assessment

The diagnosis of CVID was confirmed by the patient’s immunological profile. The patient showed mild lymphopenia with a global decrease and altered distribution of the B- and T-cell compartment already at disease onset and during follow-up as compared with age- and gender-matched healthy donors (HC) (**Table 1**).

**Table 1:** Immunological phenotyping of B and T cells, autoantibodies titres, and analysis of cytokine production by FC.

		1-FU	2-FU	3-FU	4-FU	5-FU	6-FU	Father	HC group
%		*B-cell phenotyping*							
B cells (CD19^+^)		2.00	–	1.19	1.77	0.50	2.1^+^	5.40 (1.15)	10.28 (3.74)
B naïve (CD19^+^CD27^-^)		94.00	–	90.20	93.60	81.94	91.6^+^	58.31 (6.76)	82.1 (73.0-87.3)
B memory (CD19^+^CD27^+^)		5.96	–	6.99	5.98	16.10	8.4^+^	35.80 (8.0)	17.0 (12.6-25.2)
Class-switched memory B cells (CD27^+^ IgM^-^ IgD^-^)			–	7.69	2.70	–	1.3^+^	73 (5.09)	46.46 (7.07)
IgM-memory B cells (CD27^+^ IgM^+^)			–	7.89	7.21	–	7.1^+^	11.15 (1.48)	20.16 (10.21)
CD38^low^CD21^low^		11.10	–	18.30	38.00	30.50	26.3^+^	9.84 (3.47)	2.42 (1.30-4.58)
Transitional (CD24^+^CD38^+^)		23.80	–	16.60	8.54	–	32.2^+^	2.08 (1.92)	7.64 (4.08-10.7)
Breg (CD27^+^CD24^+^)		–	–	–	6.0	5.4	–	40.2 (6.79)	36.3 (12.30)
%		*Autoantibodies*							
IAA		0.06	0	0*	0*	0	0	0.00 (0.00)	0-0.2336
GADA		14.78	2.61	2.66*	3.62*	3.23	1.23	0.02 (0.005)	0-0.8761
IA-2A		48.30	55.87	31.72*	18.89*	18.93	11.72	0.06 (0.015)	0-0.9793
ZnT8A		379.49	26.71	12.52*	3.57*	1.32	2.49	0.42 (0.25)	0-2.5091
%		*T-cell phenotyping*							
CD3^+^		79.2	75.4	81.3	47.1	79.7	81.6	70.35 (5.06)	39.2 (8.3)
CD3^+^CD4^+^		38.1	38.7	38.6	47.8	46.2	57.1	40.9 (5.45)	75.9 (11.8)
cTfh (CXCR5^+^FoxP3^-^)		39.20	34.20	18.60	30.40	34.40	7.56	6.4 (5.87)	10.85 (8.35-12.60)
cTfr (CXCR5^+^FoxP3^+^)		3.13	3.76	4.24	2.66	2.44	0.63	0.69 (0.63)	1.62 (0.97-2.18)
cTreg (CXCR5^-^FoxP3^+^)		2.67	2.43	5.22	4.26	3.39	3.46	7.65 (5.78)	4.4 (3.12-5.68)
Tfh1 (CXCR3^+^CCR6^-^)		63.90	53.90	58.60	62.10	75.40	52.00	33.83 (7.63)	26.75 (5.90)
Tfh2 (CXCR3^-^CCR6^-^)		19.40	32.40	34.10	26.70	17.10	25.80	28.93 (8.31)	36.47 (8.04)
Tfh17 (CXCR3^-^CCR6^+^)		8.29	7.83	4.64	5.05	2.65	8.22	27.33 (9.95)	26.06 (5.04)
PD1 (CD4^+^CXCR5^+^)		70.00	57.30	52.10	66.10	69.30	54.10	13.87 (4.75)	21.30 (16.40-25.30)
ICOS (CD4^+^CXCR5^+^)		6.15	5.12	4.11	1.83	1.41	4.22	0.39 (0.18)	1.45 (0.91-2.32)
CXCR3^+^PD1^-^ (CD4^+^CXCR5^+^)		22.40	23.80	7.01	12.70	13.20	–	12.31 (13.22)	8.17 (5.1)
%		*Cytokine production FC-analysis*							
CXCR5^+^	IFN-γ^+^	–	–	–	2.21	1.99	2.72	3.22 (1.21)	9.51 (10.88)
	IL-17^+^	–	–	–	2.09	0.51	0.80	1.26 (1.04)	5.06 (6.18)
	IL-21^+^	–	–	–	3.49	3.43	5.53	2.13 (0.87)	8.98 (12.07)
CXCR5^-^	IFN-γ^+^	–	–	–	10.20	13.50	25.80	11.01 (5.34)	3.69 (2.86)
	IL-17^+^	–	–	–	0.92	0.21	1.34	1.32 (1.57)	1.14 (0.37)
	IL-21^+^	–	–	–	6.17	15.10	20.07	2.8 (1.61)	3.32 (2.06)
*ng/ml*		*IgM and IgG production assay*							
CVID BM + CVID cTfh	IgM	7.78	8.35	–	–	–	–	–	1.37 (0.84)
	IgG	0.3	ND	–	–	–	–	–	9.77 (3.76)
CVID BN + CVID cTfh	IgM	1.1	0.81	–	–	ND	–	–	1.22 (1.05)
	IgG	ND	ND	–	–	ND	–	–	5.52 (3.68)

Available measurements for the index patient, for the father and for the HC pool (B and T cell phenotyping, HC n = 85; cytokine production FC-analysis, HC n = 65; IgM and IgG production assay, HC n = 16; autoantibodies, HC = internal laboratory reference) are included in the table as mean (SD) or median (IQR). The detection of autoantibodies was performed as previously described (24, 25). ^+^Values were determined in June 2022; *titres have been determined in serum samples; ND = undetermined.

By assessing the expression of CXCR5 and FoxP3 among CD3^+^CD4^+^ cells, the frequency of Circulating T follicular helper cell (cTfh) (CXCR5^+^FoxP3^-^), Circulating T follicular regulatory cell (cTfr) (CXCR5^+^FoxP3^+^), and cT_reg_ (CXCR5^-^FoxP3^+^) cells was determined. While cTfr cell frequencies in the patient were within the normal range, cT_reg_ cell frequencies were within the lower range at first but returned to average normal values in subsequent FUs (CVID cTfr, 1-FU = 3.13%; 2-FU = 3.76%; 3-FU = 4.24%; 4-FU = 2.66%; 5-FU = 2.44%; 6-FU = 0.63% vs. HC median, IQR = 1.62, 0.97–2.18, *n* = 80) (CVID cT_reg_, 1-FU = 2.67%; 2-FU = 2.43%; 3-FU = 5.22%; 4-FU = 4.26%; 5-FU = 3.39%; 6-FU = 3.46% vs. HC median, IQR = 4.4, 3.12–5.68) (**Figures 2C**, **S2A**, **Table 1**). T_reg_ cells (CD25^+^CD127^-^/loFoxP3^+^) and FoxP3 levels (γMFI) in the proband were reduced at disease (**Figures 2G–I**).

cTfh cells, on the other hand, were elevated at the onset but declined in year 5 of FU (**Table 1**). Further analyses on cTfh cell subset distribution and activation status identified a remarkable shift toward Tfh1 (CXCR3^+^CCR6^-^) cells at the expense of the Tfh2 and Tfh17 subsets (CXCR3^+^CCR6^-^ and CXCR3^-^CCR6^+^, respectively) (**Figure 2D**) that was maintained throughout the 5-year FU (**Figure S2B**, **Table 1**). Moreover, the frequency of PD-1^+^ cTfh cells was substantially higher and remained elevated over time in comparison with HC (median, IQR = 21.30, 16.40–25.30), whereas ICOS^+^ cTfh cell frequency remained higher since disease onset (~4 times higher than the control, 6.15% vs. HC median, IQR = 1.45%, 0.91–2.32) (**Figure 2E**, **Table 1**). Additionally, higher levels of plasma CXCL13, a GC blood biomarker, were observed during the 5-year FU (**Table 1**, **Figure 2F**). The father had normal frequencies and subset distribution of follicular T cells (**Table 1**).

**Figure 3:**
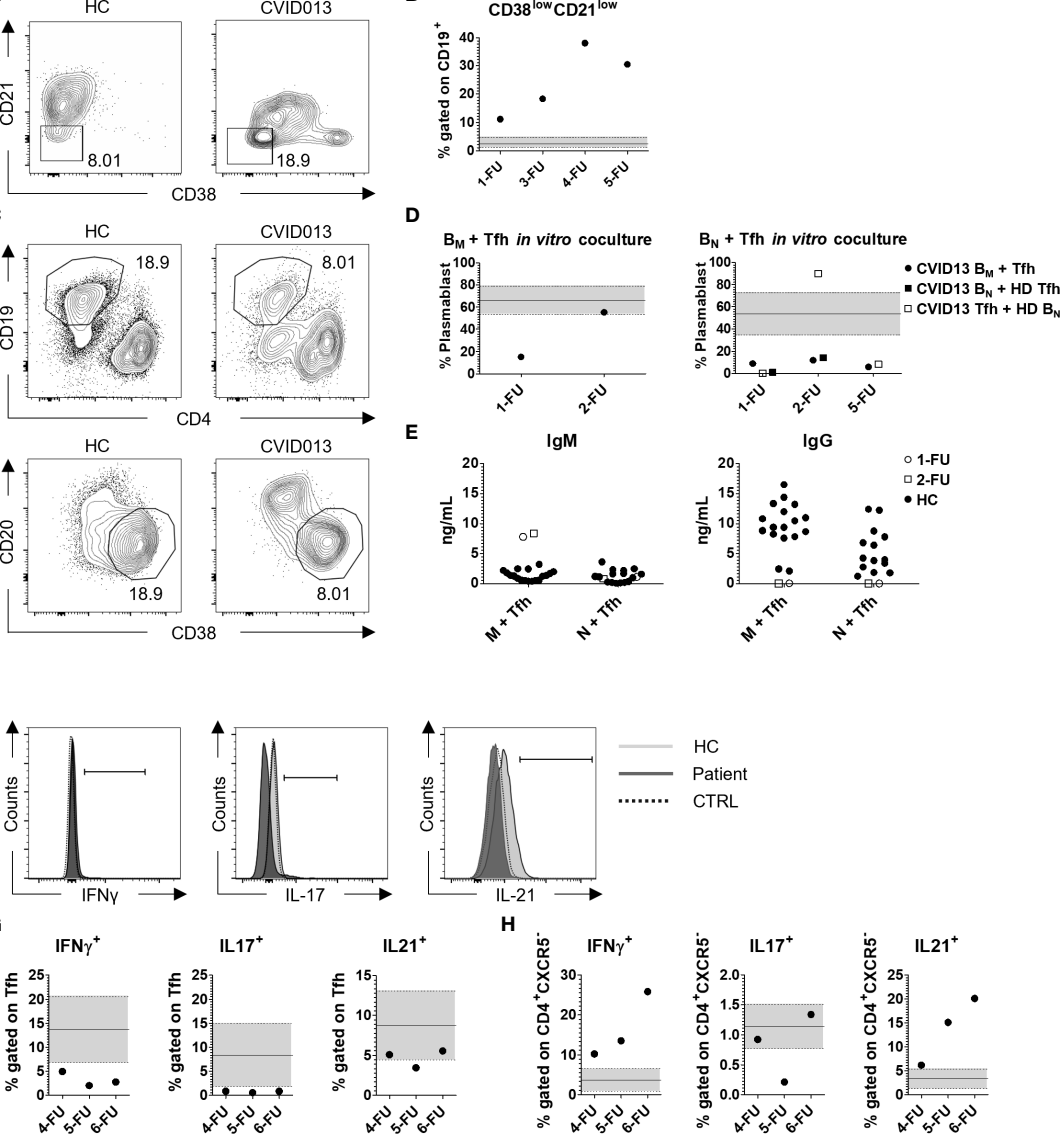
Functional analysis of B- and T-cell subsets. **(A)** Representative gating strategy for CD38^low^CD21^low^ autoreactive B cells, gated on CD19^+^ cells and **(B)** their frequency over time. CD38^low^CD21^low^ cell percentage was higher compared with the HC median, IQR (2.42%, 1.3–4.58; *n* = 85), increasing from 11.10% at the first follow-up up to 38.00% in 2020, and decreasing to 30.50% in the last monitoring. **(C, D)** Functional analysis of IgM and IgG production. Sorted B memory or B naïve cells were co-cultured with Tfh cells (1:1 ratio) in autologous (solid dot) or heterologous settings (CVID B cells with HC Tfh, solid square, or HC B cells with CVID Tfh, clear square), and the percentage of CD38^+^CD20^-^ was analyzed within CD19^+^CD4^-^ cells after 1 week. The black continuous line is representative for the mean HC percentage value ± *SD* (66.32% ± 12.46, *n* = 16) represented by the light gray area within the two dashed lines. The production of IgM and IgG was evaluated in the supernatant **(E,** **Table 1** **)**. The white dots and squares are representative for the 1-FU and 2-FU, respectively, whereas the black dots represent the HC. **(F, G)** Evaluation of IFN-γ, IL-17, and IL-21 production in CD4^+^CXCR5^+^ cells after 2-h stimulation with PMA/Ionomycin. The HC and patient slopes are identified with the light and dark gray, respectively, whereas the unstimulated control is represented by the dashed line. IFN-γ and IL-17 production was lower compared with the HC (IFN-γ mean ± *SD* = 16.66% ± 6.84; IL-17 mean ± *SD* = 8.35% ± 6.63; *n* = 65), whereas IL-21 production was lower than HC mean and comprised within the *SD* (IL-21 mean ± *SD* = 8.74% ± 4.30; *n* = 65). **(H)** IFN-γ, IL-17, and IL-21 production in CD4^+^CXCR5^-^ cells after 2-h stimulation with PMA/Ionomycin. IFN-γ and IL-21 production was higher compared with the HC (IFN-γ mean ± *SD* = 3.69% ± 2.86; IL-21 mean ± *SD* = 3.32% ± 2.06; *n* = 65), whereas IL-17 production was comparable with HC (IL-17 mean ± *SD* = 1.14% ± 0.37; *n* = 65). .

The percentage of total CD19^+^ B cells was low during the 5-year follow-up (FU) (CVID B cells, 1-FU = 2%; 3-FU = 1.19%; 4-FU = 1.77%; 5-FU = 0.5%; 6-FU = 2.1% vs. HC mean ± *SD* = 10.28 ± 3.74, *n* = 90). The frequency of B memory cells (CD19^+^CD27^+^) was lower than HC (median, IQR = 17.0, 12.6–25.2). Potentially autoreactive B cells defined as CD19^+^CD21^low^CD38^low^ B cells (**Figure 3A**) were present at higher frequency in the index patient at diagnosis as compared with HC (CVID013 = 11.1% vs. HC median, IQR = 2.42%, 1.30–4.58) and increased over time (3-FU = 18.30%; 4-FU = 38.00%; 5-FU = 30.50%; 6-FU = 26.30%) (**Figure 3B**, **Table 1**). In contrast to the index patient, circulating B cell frequency and subset distribution in the father were normal (**Table 1**).

To assess the functionality of B and Tfh cells, we performed *in vitro* B-cell helper assay. FACS-sorted memory and naïve B cells were co-cultured with cTfh cells in autologous (CVID B cells with CVID Tfh cells) and heterologous settings (CVID B cells with HC Tfh cells or *vice versa*) and the percentage of CD20^-^CD38^+^ plasmablasts together with IgM and IgG levels were evaluated after a week (**Figures 3C–E**). Due to technical constraints, we were able to perform the assay at three FU. The percentage of plasmablast differentiating from memory B cells in the presence of autologous Tfh cells was lower with respect to HC at diagnosis but improved in 2-FU (**Figure 3D**, left panel). Patient Tfh cells were able to induce the production of IgM by autologous B memory cells at levels that were higher than the control co-cultures, (1-FU = 7.78ng/ml; 2-FU = 8.35ng/ml vs. HC mean ± *SD* = 1.37ng/ml ± 0.84, *n* = 18). On the other hand, IgM production by B naïve cells was similar to HC (1-FU = 1.1ng/ml; 2-FU = 0.80ng/ml vs. HC mean ± *SD* = 1.22 ng/ml ± 1.05, *n* = 16). Tfh cells co-cultured either with autologous or heterologous B naïve cells were unable to induce class switching and IgG production *in vitro* (**Figures 3D, E**).

Tfh (CD4^+^CXCR5^+^) and non-Tfh (CD4^+^CXCR5^-^) cell functional status was also evaluated *in vitro* by intracellular cytokine profile. Total PBMCs were activated with PMA/Ionomycin and the expression of interferon-γ (IFN-γ), interleukin-17 (IL-17), and IL-21 was evaluated by flow cytometry (FC) (**Figures 3F, G**). Within the CXCR5^+^ compartment, IFN-γ and IL-17 producing cells were fewer compared with HC (**Table 1**). Also, IL-21 production was lower than HC (CVID013 IL-21 range = 3.43% - 5.53% vs. HC IL-21 mean ± *SD* = 8.74% ± 4.30; *n* = 65). On the contrary, higher frequencies of IFN-γ and IL-21 producing cells were observed within the CXCR5^-^ compartment (**Table 1**, **Figure 3H**).

## Discussion

This case report describes a patient diagnosed with ITP, CVID, and T1D with a monoallelic mutation in *BAFFR* (H159Y) inherited from the father. Two years after CVID and T1D stage 2 diagnosis, RTX was administered to treat peripheral polyneuropathy with a potentially positive impact on diabetes progression. Additional diet adjustment (hypoglycemic/ketogenic) led to an 8-kg weight loss that possibly impacted the disease course. Eventually, the patient progressed to insulin dependency, despite a decline in islet AAbs levels. The patient displayed the typical immunological signs of CVID, that is, reduction in circulating B cells, switched memory B cells, and an increase in autoreactive CD21^low^CD38^low^ B cells. B cell counts remained low during a 5-year FU. The patient was positive for SARS-CoV-2 antibodies prior to infection and vaccination, probably secondary to the presence of these antibodies in IVIg. After receiving three vaccine doses and natural SARS-CoV-2 infection, his anti–SARS-CoV-2 antibodies remained detectable. Generally, CVID patients, especially those with autoimmunity, have variable alterations in humoral responses against vaccines, including against SARS-CoV-2, that could account for a low specific response to some infections and vaccination (23). Interestingly, B naïve and memory subset frequencies increased over time but remained reduced and even declined in absolute numbers. When cultured *in vitro* with autologous and heterologous Tfh cells derived from HC, memory B cells were able to produce IgM, whereas IgG production was compromised, suggesting dysfunctional B and/or Tfh cells.

cTfh cells were present at elevated frequencies during the first 4 years of FU and produced reduced amounts of IFN-γ and IL-21 when challenged *in vitro*. cTfh cells showed a shift toward a Tfh1 phenotype accompanied by an increase in activation markers PD-1 and ICOS. cTfh cell activation status was reflected in the blood where elevated plasmatic concentrations of CXCL13 were found (26). Interestingly, IL-21 production by CXCR5^-^ CD4^+^ cells was highly elevated when compared with HC. Given the connection between IL-21 production and T1D (27), elevated IL-21 production by CXCR5^-^CD4^+^ T cells could have influenced T1D development in the index patient.

BAFFR is essential for B-cell development, and reduced BAFFR expression or signaling, as in BAFFR deficiency, leads to decreased B cell survival and hypogammaglobulinemia (28). BAFFR can be expressed on the surface of activated T cells including T_regs_ albeit at low levels (29–32). By re-analyzing our previously published RNA-seq data in sorted Tfh cells from the index patient (CVID013) (19), BAFFR mRNA levels were elevated as compared with controls (**Figure S3**). However, at a protein level, Tfh cells expressed slightly reduced BAFFR levels on their cell surface. The BAFFR H159Y mutation identified in the patient has been previously associated not only with autoimmune diseases, such as systemic lupus erythematosus, multiple sclerosis, and Sjogren’s syndrome, but also in non-Hodgkin’s lymphoma (33). It is currently unknown how this variant affects protein trafficking, signaling, and degradation. Previous studies have shown that it increases TRAF2, TRAF3, and TRAF6 recruitment to BAFFR, potentiating NF-κB1 and NF-κB2 activity and immunoglobulin production in B cells (28, 33–38). According to our RNA-seq data, BAFFR-mediated dysregulation affected Tfh cell cycle, T-cell activation, and proliferation pathways, and altered the expression of genes involved in signal transduction, apoptosis, and Tfh identity (i.e., BCL-6) (**Figures S4–S6**). On the other hand, the UV response pathway was down-regulated including pathways involved in apoptosis, cell cycle, proliferation, and immune functions (promoting proliferation) (**Figures S4–S7**) (19). Further analyses are required to determine the functional role of H159Y in human Tfh cells and B cells and their contribution to CVID and T1D development.

The H159Y variant has been previously described in association with another polymorphism, P21R, which has been described in some patients with CVID (37). These patients displayed lower B cell numbers due to reduced BAFFR expression levels. Possibly, other genetic variants in BAFFR or in other genes related to this pathway are present and contributed to the clinical course of CVID and T1D in the index patient. Of note, the patient’s father is affected by autoimmune thyroiditis and has no T1D nor CVID despite having the same BAFFR mutation and reduced surface BAFFR levels on his B cells. Thus, incomplete disease penetrance might underlie the discrepancies between father and son, similarly to previous CVID reports where family members carry the same heterozygous mutation (34).

Given the absence of a T1D-HLA risk, alterations in BAFFR and humoral dysregulation might have led to T1D. In contrast to other autoimmune diseases, for example, SLE, where BAFF–BAFFR signalling has been extensively studied, limited studies have been conducted in T1D. In one of such studies, reduced BAFFR levels on circulating B cells were observed in patients with longstanding T1D (39). Given the 6-year time window from the time of stage 2 T1D to stage 2 T1D diagnosis, we speculate that BAFFR humoral dysregulation contributed to T1D with slow kinetics or, perhaps, RTX and IVIg therapy delayed the disease onset.

The effect of IVIg therapy in B cells seems to be rather complex and not well understood (40), and there is not enough evidence supporting a beneficial role of IVIg in T1D progression. In the index patient, the treatment did not alter circulating B-cell frequency over the 6 years follow-up and did not affect B-cell ability to stimulate IgM production *in vitro*. It is possible that the alterations in B-cell subset composition were partly mediated by IVIg and could have affected T1D progression, possibly by AAb dilution or by affecting autoreactive B-cell frequency (41). Tfh were able to stimulate the production of IgM but no IgG in B-cell co-cultures *in vitro*; however, we did not explore the possibility that the patient had less class-switched IgG^+^ memory B cells explaining our *in vitro* B cell help findings. Additional experiments with sorted IgM^+^ vs. IgM^-^ memory B cells will be necessary to clarify this point.

Belimumab, the human monoclonal antibody that blocks BAFF, is currently employed for the treatment of persistently active systemic lupus erythematosus (33) BAFFR blockade in murine models of T1D was also shown to protect from disease development, a mechanism that involved Breg induction (42). RTX depletes B cells and was shown to preserve C-peptide levels in patients with new-onset T1D (43). The index patient received RTX treatment 2 years after stage 2 T1D diagnosis and 3 years later; after partial B-cell reconstitution, he progressed to insulin-dependent T1D. In the NOD model of T1D, no synergy between RTX and anti-BAFFR mAb treatment was seen as RTX eliminated anti-BAFFR–induced Bregs (42). It remains unknown the effect of RTX on Bregs in the index patient, but possibly RTX did not aggravate disease progression but was rather beneficial.

Despite several weaknesses emanating from the study of a single case and the lack of studies of BAFFR signaling, our data suggest a possible involvement of the BAFFR H159Y variant in T1D pathogenesis and suggest that the BAFF/BAFFR axis might be a target of interest for the pharmacological modulation of T1D.

## Data availability statement

The original contributions presented in the study are included in the article/**Supplementary Material**. Further inquiries can be directed to the corresponding authors.

## Ethics statement

The studies involving human participants were reviewed and approved by Ethical Committee of HSR (Tiget06, Tiget09 and DRI004 protocols). Written informed consent to participate in this study was provided by the participants’ legal guardian/next of kin.

## Author contributions

BL and LPac contributed equally. Conception and design: BL, LPac, CCa, and GF. Development of methodology: BL, LPac, and GM. Acquisition of data: BL, LP, GM, TJ, SC, JG, IMa, IMe, MB, EZ, BR, CCi, FB, CG, PZ, NR, AD, GA, LPas, PC, PP, AF, VL, PC, and RS. Analysis and interpretation of data: BL, LPac, SG, MPC, GDM, AA, LPi, CCa, and GF. Writing, review, and revision of the manuscript: BL, LPas, SG, GA, LPa, MPC, GDM, AA, LPi, CCa, and GF. Study supervision: GF. All authors contributed to the article and approved the submitted version.

## Funding

This work was supported from 5x1000 OSR pilot & seed grant and GR-2016-02365089 to GF and MPC.

## Conflict of interest

The authors declare that the research was conducted in the absence of any commercial or financial relationships that could be construed as a potential conflict of interest.

## Publisher’s note

All claims expressed in this article are solely those of the authors and do not necessarily represent those of their affiliated organizations, or those of the publisher, the editors and the reviewers. Any product that may be evaluated in this article, or claim that may be made by its manufacturer, is not guaranteed or endorsed by the publisher.

## Abbreviations

AAb, Autoantibody; AITP, Autoimmune thrombocytopenic purpura; BAFFR, B-cell activating factor receptor; cTfh, Circulating T follicular helper cell; cTfr, Circulating T follicular regulatory cell; cTreg, Circulating T regulatory cell; CVID, Common variable immunodeficiency; FACS, Fluorescence activated cell sorter; FC, Flow cytometry; FMO, Fluorescence minus one; FPG, Fasting plasma glucose; FU, Follow-up, GADA, Glutamic acid decarboxylase antibodies; GRS, Genetic risk score; HC, Healthy control; HLA, Human leukocyte antigen; I-A2, Insulinoma-associated antigen 2; IAA, Insulin autoantibodies; IQR, Interquartile range; ITP, Immune thrombocytopenia; MFI, Median fluorescence intensity; PBMCs, Peripheral blood mononucleated cells, SD, Standard deviation; SNP, Single-nucleotide polymorphism; T1D, Type 1 diabetes; WES, Whole exome sequencing, ZnT8, Zinc transporter 8.

## References

[1] Hinman RM Cambier JC. Role of b lymphocytes in the pathogenesis of type 1 diabetes. Curr Diabetes Rep (2014) 14(11) 543. doi: 10.1007/s11892-014-0543-8 25189436

[2] Edner NM Heuts F Thomas N Wang CJ Petersone L Kenefeck R. Follicular helper T cell profiles predict response to costimulation blockade in type 1 diabetes. Nat Immunol (2020) 21(10) 1244–1255. doi: 10.1038/s41590-020-0744-z PMC7610476 32747817

[3] Gardner G Fraker CA. Natural killer cells as key mediators in type I diabetes immunopathology. Front Immunol (2021) 12. doi: 10.3389/fimmu.2021.722979 PMC8417893 34489972

[4] Jia X Gu Y High H Yu L. Islet autoantibodies in disease prediction and pathogenesis. Diabetol Int (2020) 11(1) 6–10. doi: 10.1007/s13340-019-00414-9 PMC6942067 31949998

[5] Fousteri G Ippolito E Ahmed R Rahim Hamad A. Beta-cell specific autoantibodies: Are they just an indicator of type 1 diabetes? Curr Diabetes Rev (2017) 13(3). 322–329 doi: 10.2174/1573399812666160427104157 PMC5266674 27117244

[6] Insel RA Dunne JL Atkinson MA Chiang JL Dabelea D Gottlieb PA. Staging presymptomatic type 1 diabetes: a scientific statement of JDRF, the endocrine society, and the American diabetes association. Diabetes Care (2015) 38(10) 1964–1974. doi: 10.2337/dc15-1419 PMC5321245 26404926

[7] Ziegler AG Rewers M Simell O Simell T Lempainen J Steck A. Seroconversion to multiple islet autoantibodies and risk of progression to diabetes in children. JAMA (2013) 309(23) 2473–2479. doi: 10.1001/jama.2013.6285 PMC4878912 23780460

[8] Todd JA. Etiology of type 1 diabetes. Immunity (2010) 32(4) 457–467. doi: 10.1016/j.immuni.2010.04.001 20412756

[9] Fousteri G Rodrigues EM Giamporcaro GM Falcone M. A machine learning approach to predict response to immunotherapy in type 1 diabetes. Cell Mol Immunol (2021) 18(3) 515–517. doi: 10.1038/s41423-020-00594-4 PMC8027390 33318626

[10] Oram RA Patel K Hill A Shields B McDonald TJ Jones A. A type 1 diabetes genetic risk score can aid discrimination between type 1 and type 2 diabetes in young adults. Diabetes Care (2016) 39(3) 337–344. doi: 10.2337/dc15-1111 PMC5642867 26577414

[11] Johnson MB Patel KA de Franco E Houghton JAL McDonald TJ Ellard S. A type 1 diabetes genetic risk score can discriminate monogenic autoimmunity with diabetes from early-onset clustering of polygenic autoimmunity with diabetes. Diabetologia (2018) 61(4) 862–869. doi: 10.1007/s00125-018-4551-0 PMC6448971 29417186

[12] Tangye SG Al-Herz W Bousfiha A Chatila T Cunningham-Rundles C Etzioni A. Human inborn errors of immunity: 2019 update on the classification from the international union of immunological societies expert committee. J Clin Immunol (2020) 40(1):24–64. doi: 10.1007/s10875-019-00737-x 31953710 PMC7082301

[13] Ochtrop MLG Goldacker S May AM Rizzi M Draeger R Hauschke D. T And b lymphocyte abnormalities in bone marrow biopsies of common variable immunodeficiency. Blood (2011) 118(2) 309–318. doi: 10.1182/blood-2010-11-321695 21576700

[14] Warnatz K Voll RE. Pathogenesis of autoimmunity in common variable immunodeficiency. Front Immunol (2012) 3. doi: 10.3389/fimmu.2012.00210 PMC3399211 22826712

[15] Taubenheim N von Hornung M Durandy A Warnatz K Corcoran L Peter HH. Defined blocks in terminal plasma cell differentiation of common variable immunodeficiency patients. J Immunol (2005) 175(8) 5498–5503. doi: 10.4049/jimmunol.175.8.5498 16210658

[16] le Saos-Patrinos C Loizon S Blanco P Viallard JF Duluc D. Functions of tfh cells in common variable immunodeficiency. Front Immunol (2020) 11. doi: 10.3389/fimmu.2020.00006 PMC7002358 32082308

[17] van de Ven AAJM Warnatz K. The autoimmune conundrum in common variable immunodeficiency disorders. Curr Opin Allergy Clin Immunol (2015) 15(6):. doi: 10.1097/ACI.0000000000000218 26485099

[18] Deenick EK Ma CS. The regulation and role of T follicular helper cells in immunity. Immunology (2011) 134(4) 361–367. doi: 10.1111/j.1365-2567.2011.03487.x PMC3230790 22043829

[19] Milardi G di Lorenzo B Gerosa J Barzaghi F di Matteo G Omrani M. Follicular helper T cell signature of replicative exhaustion, apoptosis and senescence in common variable immunodeficiency. Eur J Immunol (2022). doi: 10.1002/eji.202149480 PMC9542315 35562849

[20] Gereige JD Maglione PJ. Current understanding and recent developments in common variable immunodeficiency associated autoimmunity. Front Immunol (2019) 10. doi: 10.3389/fimmu.2019.02753 PMC6914703 31921101

[21] Milota T Šumník Z Obermannová B Králíčková P Vondrák K Klocperk A. Negativity for specific autoantibodies in patients with type 1 diabetes that developed on a background of common variable immunodeficiency. Int Arch Allergy Immunol (2015) 168(3) 197–204. doi: 10.1159/000441723 26796963

[22] Bayram RO Özdemir H Emsen A Turk Dagi H Artaç H. Reference ranges for serum immunoglobulin (IgG, IgA, and IgM) and IgG subclass levels in healthy children. Turkish J Med Sci (2019) 49(2):497–505. doi: 10.3906/sag-1807-282 PMC7018341 30997788

[23] Amodio D Ruggiero A Sgrulletti M Pighi C Cotugno N Medri C. Humoral and cellular response following vaccination with the BNT162b2 mRNA COVID-19 vaccine in patients affected by primary immunodeficiencies. Front Immunol (2021) 12. doi: 10.3389/fimmu.2021.727850 PMC8521226 34671350

[24] Lampasona V Passerini L Barzaghi F Lombardoni C Bazzigaluppi E Brigatti C. Autoantibodies to harmonin and villin are diagnostic markers in children with IPEX syndrome. PloS One (2013) 8(11):e78664. doi: 10.1371/journal.pone.0078664 24250806 PMC3826762

[25] Liberati D Wyatt RC Brigatti C Marzinotto I Ferrari M Bazzigaluppi E. A novel LIPS assay for insulin autoantibodies. Acta Diabetol (2018) 55(3):263–70. doi: 10.1007/s00592-017-1082-y 29305766

[26] Havenar-Daughton C Lindqvist M Heit A Wu JE Reiss SM Kendric K. CXCL13 is a plasma biomarker of germinal center activity. Proc Natl Acad Sci (2016) 113(10) 2702–2707. doi: 10.1073/pnas.1520112113 PMC4790995 26908875

[27] Long D Chen Y Wu H Zhao M Lu Q. Clinical significance and immunobiology of IL-21 in autoimmunity. J Autoimmun (2019) 99 1–14. doi: 10.1016/j.jaut.2019.01.013 30773373

[28] Warnatz K Salzer U Rizzi M Fischer B Gutenberger S Böhm J. B-cell activating factor receptor deficiency is associated with an adult-onset antibody deficiency syndrome in humans. Proc Natl Acad Sci (2009) 106(33) 13945–13950. doi: 10.1073/pnas.0903543106 PMC2722504 19666484

[29] Ng LG Sutherland APR Newton R Qian F Cachero TG Scott ML. B cell-activating factor belonging to the TNF family (BAFF)-r is the principal BAFF receptor facilitating BAFF costimulation of circulating T and b cells. J Immunol (2004) 173(2):. doi: 10.4049/jimmunol.173.2.807 15240667

[30] Hu S Wang R Zhang M Liu K Tao J Tai Y. BAFF promotes T cell activation through the BAFF-BAFF-R-PI3K-Akt signaling pathway. Biomed Pharmacother (2019) 114 108796. doi: 10.1016/j.biopha.2019.108796 30921706

[31] Mackay F Leung H. The role of the BAFF/APRIL system on T cell function. Semin Immunol (2006) 18(5) 284–289. doi: 10.1016/j.smim.2006.04.005 16931039

[32] Ye Q Wang L Wells AD Tao R Han R Davidson A. BAFF binding to T cell-expressed BAFF-r costimulates T cell proliferation and alloresponses. European J Immunol (2004) 34(10):. doi: 10.1002/eji.200425198 15368291

[33] Hildebrand JM Luo Z Manske MK Price-Troska T Ziesmer SC Lin W. A BAFF-r mutation associated with non-Hodgkin lymphoma alters TRAF recruitment and reveals new insights into BAFF-r signaling. J Exp Med (2010) 207(12):2569–79. doi: 10.1084/jem.20100857 PMC2989778 21041452

[34] Losi CG Silini A Fiorini C Soresina A Meini A Ferrari S. Mutational analysis of human BAFF receptor TNFRSF13C (BAFF-r) in patients with common variable immunodeficiency. J Clin Immunol (2005) 25(5) 496–502. doi: 10.1007/s10875-005-5637-2 16160919

[35] Pieper K Rizzi M Speletas M Smulski CR Sic H Kraus H. A common single nucleotide polymorphism impairs b-cell activating factor receptor’s multimerization, contributing to common variable immunodeficiency. J Allergy Clin Immunol (2014) 133(4):. doi: 10.1016/j.jaci.2013.11.021 24406071

[36] Germinaro M Reynolds P Knight V Alam R. Association of b-cell activating factor receptor deficiency with the P21R polymorphism and common variable immunodeficiency. Ann Allergy Asthma Immunol (2015) 115(1) 82–83. doi: 10.1016/j.anai.2015.04.020 PMC4760690 26012370

[37] Lougaris V Baronio M Moratto D Cardinale F Plebani A. Monoallelic BAFFR P21R/H159Y mutations and familiar primary antibody deficiencies. J Clin Immunol (2016) 36(1):1–3. doi: 10.1007/s10875-015-0217-6 26613719

[38] Yang S Li JY Xu W. Role of BAFF/BAFF-r axis in b-cell non-Hodgkin lymphoma. Crit Rev Oncol/Hematol (2014) 91(2) 113–122. doi: 10.1016/j.critrevonc.2014.02.004 24629840

[39] Parackova Z Klocperk A Rataj M Kayserova J Zentsova I Sumnik Z. Alteration of b cell subsets and the receptor for b cell activating factor (BAFF) in paediatric patients with type 1 diabetes. Immunol Lett (2017) 189:94–100. doi: 10.1016/j.imlet.2017.04.009 28414179

[40] Quinti I Mitrevski M. Modulatory effects of antibody replacement therapy to innate and adaptive immune cells. Front Immunol (2017) 8. doi: 10.3389/fimmu.2017.00697 PMC5472665 28670314

[41] Mitrevski M Marrapodi R Camponeschi A Lazzeri C Todi L Quinti I. Intravenous immunoglobulin replacement therapy in common variable immunodeficiency induces b cell depletion through differentiation into apoptosis-prone CD21low b cells. Immunol Res (2014) 60(2–3):330–8. doi: 10.1007/s12026-014-8599-8 25407649

[42] Wang Q Racine JJ Ratiu JJ Wang S Ettinger R Wasserfall C. Transient BAFF blockade inhibits type 1 diabetes development in nonobese diabetic mice by enriching immunoregulatory b lymphocytes sensitive to deletion by anti-CD20 cotherapy. J Immunol (2017) 199(11) 3757–3770. doi: 10.4049/jimmunol.1700822 PMC5698153 29055002

[43] Pescovitz MD Greenbaum CJ Bundy B Becker DJ Gitelman SE Goland R. B-lymphocyte depletion with rituximab and β-cell function: Two-year results. Diabetes Care (2014) 37(2):453–9. doi: 10.2337/dc13-0626 PMC3898764 24026563

[44] Shrestha S Budhathoki P Adhikari Y Marasini A Bhandari S Mir WAY. Belimumab in lupus nephritis: A systematic review and meta-analysis. Cureus (2021). doi: 10.7759/cureus.20440 PMC8760003 35047277

