# Supplementary material for: Natural history of type 1 diabetes on an immunodysregulatory background with genetic alteration in B-cell activating factor receptor: A case report
Source: Front Immunol. 2022 Aug 26;13:952715. doi: 10.3389/fimmu.2022.952715 (PMC9459137; doi:10.3389/fimmu.2022.952715)
Supplement: Supplementary file 1 [file DataSheet_1.docx]

**Materials and Methods**

***Study conduction***

Collection of blood samples was performed after the subjects or parents’ signature of informed consent for biological sample collection, including genetic analyses, in the context of protocols approved by the Ethical Committee of HSR (Tiget06, Tiget09 and DRI004 protocols). The case report was written following the CARE - Checklist of information to include when writing a case report (https://www.care-statement.org/checklist).

***WES and BAFFR mutation sequencing***

WES was performed by Genomnia (http://www.genomnia.com/). DNA libraries were sequenced on a Hiseq 4000 (Illumina) for paired-end 150 bp reads. Sequencing reads were mapped to the reference human genome (UCSC hg19 and hg38) with the Torrent Suite (5.10.0). The bam files generated from two chips were merged with the Combine Alignments utility of the Torrent Suite. The samples were analyzed with the workflow Ion Report Ampliseq Exome Single Sample (germline) version 5.6. The quality of the sequencing was verified with the fastqc software v.0.10.1 e samstat v.1.08.

Candidate variants responsible for the disease were analyzed highlighting those that fulfilled the following criteria:

- Quality > 40 (to exclude false positives)

- Minor allele frequency MAF < 1% (“rare variants”), or < 5% (“uncommon variants”)

- Variants of candidate genes

- Non-synonymous exonic variants.

BAFFR mutation was validated by Sanger sequencing after amplification using the following primers: 5’ GAGCTGAATTTGATTTCCAAGC; 3’ TGAGTACGGAGCCTCTACCC. Amplified DNA fragments were purified using QIAquick PCR Purification Kit (QIAGEN), according to the manufacturer’s instructions. At the end of the purification 400 ng of DNA were sent to sequence with the Sanger method to Eurofins Scientific. The electropherogram was analyzed using FinchTV program and Nucleotide BLAST.

***T1D GRS***

We calculated the T1D GRS by typing 30 common genetic variants associated with T1D, as previously described (10). The DNA was extracted from saliva or peripheral blood samples, using the QIAamp DNA Mini and Blood Mini Kit (Qiagen, Hilden, Germany) or a Promega-Maxwell automatic extractor, following the manufacturer’s instructions. The analysis of 4 SNPs associated to the HLA risk haplotypes DR3 and DR4-DQ8 was performed by droplet digital PCR (ddPCR) (Bio-Rad, Hercules, CA, USA). The remaining 26 SNPs located in other susceptibility genes were typed using the high-throughput OpenArray system (Life Technologies, Carlsbad, CA, USA) (31). The calculation of the score generated by summing the effective allele dosage of each variant multiplied by the natural log (ln) of the odds ratio was performed as previously described (28).

***Autoantibody detection***

The detection of autoantibodies was performed as previously described (43-44).

***Flow cytometry (FC) analyses***

FC analyses were performed on peripheral blood mononuclear cells (PBMC) isolated from heparinized blood by Ficoll density gradient centrifugation (Lymphoprep, Stemcell), and whole blood (EDTA). PBMC were stained for the surface markers CD45RA (HI100), CD4 (VIT4), CD25 (2A3), PD-1 (eBioJ105), CD3 (BW264/56), CXCR5 (J252D4), CD19 (SJ25C1), CD14 (TUK4), CD8 (BW135/80), CD27 (M-T27), CD19 (SJ25C1), CD21 (B-LY4), CD38 (HIT2), CD24 (SN3), IgD (IA6-2), IgM (G20-127), IgG (polyclonal), IgA (polyclonal), BAFFR (11C1). For the analysis of FoxP3, PBMC were fixed and permeabilized for intracellular staining with the FoxP3/Transcription Factor Staining Buffer Set (eBioscience) prior to staining with FoxP3 (259D). Whole blood cells (EDTA) were stained with no prior isolation with CD45RA (T6D11), CD4 (REA623), CCR6 (G034E3), CXCR3 (1C6), ICOS (ISA-3), CD3 (BW264/56), CXCR5 (J252D4), CD45 (HI30), PD-1 (J43), (VIT4). The complete list of antibody combinations and staining is available in Supplementary Table 1. Samples were analyzed on BD FACSCanto™ II.

***In vitro IgG and IgM production assay***

B memory, B naïve, and Tfh cells were sorted by FACS using an antibody panel described in Supplementary Table 1. Sorted cells (BD FACSAria Fusion) were cultured in 10% RPMI with Staphylococcal enterotoxin B (SEB) (S4881, SIGMA) at 37°C 5% vol CO_2_ for 1 week as follow: B_M_ + Tfh cells, B_N_ + Tfh cells in a 1:1 ratio. Cells were then stained for CD4 (SK3), CD19 (SJ25C1), CD20 (REA780), CD38 (HIT2), and the supernatant was used to evaluate IgG and IgM production by ELISA (Human IgG Total Uncoated ELISA kit and Human IgM Total Uncoated ELISA kit E-Bioscience) following manufacturer’s instructions.

***CXCL13 Plasma Evaluation***

CXCL13 was evaluated in plasma EDTA by ELISA (Human CXCL13/BLC/BCA-1 Quantikine® ELISA Kit, R & D Systems) following manufacturer’s instructions. To collect plasma, whole PB (EDTA) was centrifuged at 1000 rpm for 15 min. Plasma was further centrifuged at 13000 rpm for 10 min to remove debris.

***RNAseq data analysis***

RNAseq data were obtained and analyzed as described in Milardi G, et al. 2022 (19). Heatmaps were generated using the R/Bioconductor package *pheatmap*. ssGSEA was computed using the R/Bioconductor package SGVA with default parameters.

**Supplementary Table 1. Antibody combinations used in the study.**

| **Immunostaining panel** | **Antibody** | **Fluorochrome** | **Clone** | **Manufacturer** |
| --- | --- | --- | --- | --- |
| cTfh/cTfr cell panel (PBMC) | FOXP3 | FITC | 259D | BioLegend |
|  | CD45RA | PE | HI100 | Miltenyi |
|  | PD-1 | PE-Cy7 | J105 | eBioscience |
|  | CD4 | PerCP | VIT4 | Miltenyi |
|  | CD25 | APC | 2A3 | BD |
|  | ICOS | PE-Cy7 | ISA-3 | eBioscience |
|  | CD3 | APC-Cy7 | BW264/56 | Miltenyi |
|  | CXCR5 | BV421 | J252D4 | BioLegend |
|  | CD19 | PO | SJ25C1 | BD Biosciences |
|  | CD14 | PO | TUK4 | Miltenyi |
|  | CD8 | PO | BW135/80 | Miltenyi |
| cTfh subsets  (whole blood) | CD45RA | FITC | T6D11 | Miltenyi |
|  | CD4 | PE | REA623 | Miltenyi |
|  | CCR6 | PerCP | G034E3 | BioLegend |
|  | CXCR3 | APC | IC6 | BD Biosciences |
|  | ICOS | PE-Cy7 | ISA-3 | Invitrogen |
|  | CD3 | APC-Cy7 | BW264/56 | Miltenyi |
|  | CXCR5 | BV421 | J252D4 | BioLegend |
|  | CD45 | PO | HI30 | BioLegend |
| Highly functional cTfh cell panel  (whole blood) | CD45RA | FITC | T6D11 | Miltenyi |
|  | CD4 | PerCP | VIT4 | Miltenyi |
|  | ICOS | PE-Cy7 | ISA-3 | eBioscience |
|  | CXCR3 | APC | IC6 | BD Biosciences |
|  | PD-1 | PE | J105 | eBioscience |
|  | CD3 | APC-Cy7 | BW264/56 | Miltenyi |
|  | CXCR5 | BV421 | J252D4 | BioLegend |
|  | CD45 | PO | HI30 | BioLegend |
| B cells (PBMC) | IgM | FITC | G20-127 | BD Biosciences |
|  | CD21 | PE | B-LY4 | BD Biosciences |
|  | CD27 | APC | M-T271 | BD Biosciences |
|  | CD38 | PerCP-Cy5.5 | HIT2 | BD Biosciences |
|  | CD19 | PE-Cy7 | SJ 25C1 | BD Biosciences |
|  | CD24 | PB | SN3 | EXBIO |
|  | IgD | BIO | IA6-2 | BD Biosciences |
|  | Streptavidin | PO | - | ThermoFischer |
| B cell - BAFFR | IgG | FITC | polyclonal | Jackson Immunoresearch |
|  | CD19 | PE | HIB19 | BD Bioscience |
|  | CD38 | PERPC | HIT2 | BioLegend |
|  | BAFFR | APC | 11C1 | BioLegend |
|  | CD21 | PECy7 | Bu32 | BioLegend |
|  | CD27 | APCh7 | M-T271 | BD Bioscience |
|  | CD24 | PB | SN3 | EXBIO |
|  | IgD-biot-strept | PO | IA6-2 | BD Bioscience |
| cTfh, B memory  and B naive  (Sorting) | CD45RA | FITC | REA1047 | Miltenyi Biotec |
|  | CD3 | PE | SK7 | BD bioscience |
|  | CD4 | PO | VIT4 | Miltenyi Biotec |
|  | CD25 | APC | BC96 | BioLegend |


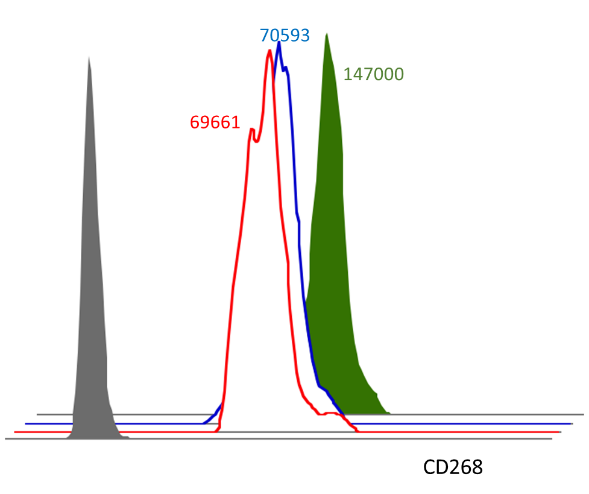

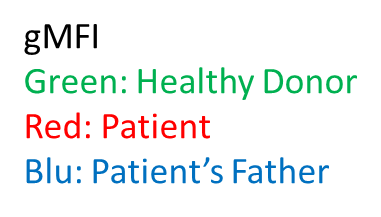


**Supplementary Figure 1.** Representative gating strategy to evaluate BAFFR distribution on B cells. Red, blue, and green slopes for index patient, the proband father and the control, respectively.

A

B

**Supplementary Figure** **2.** cTfh, cTfr, and cTreg distribution (A) and cTfh subset distribution (B) during the 6 follow-ups. Solid dots and squares represent the index patient and the proband father, respectively. HC group mean is represented by the continuous line within the light gray area, representing the standard deviation.


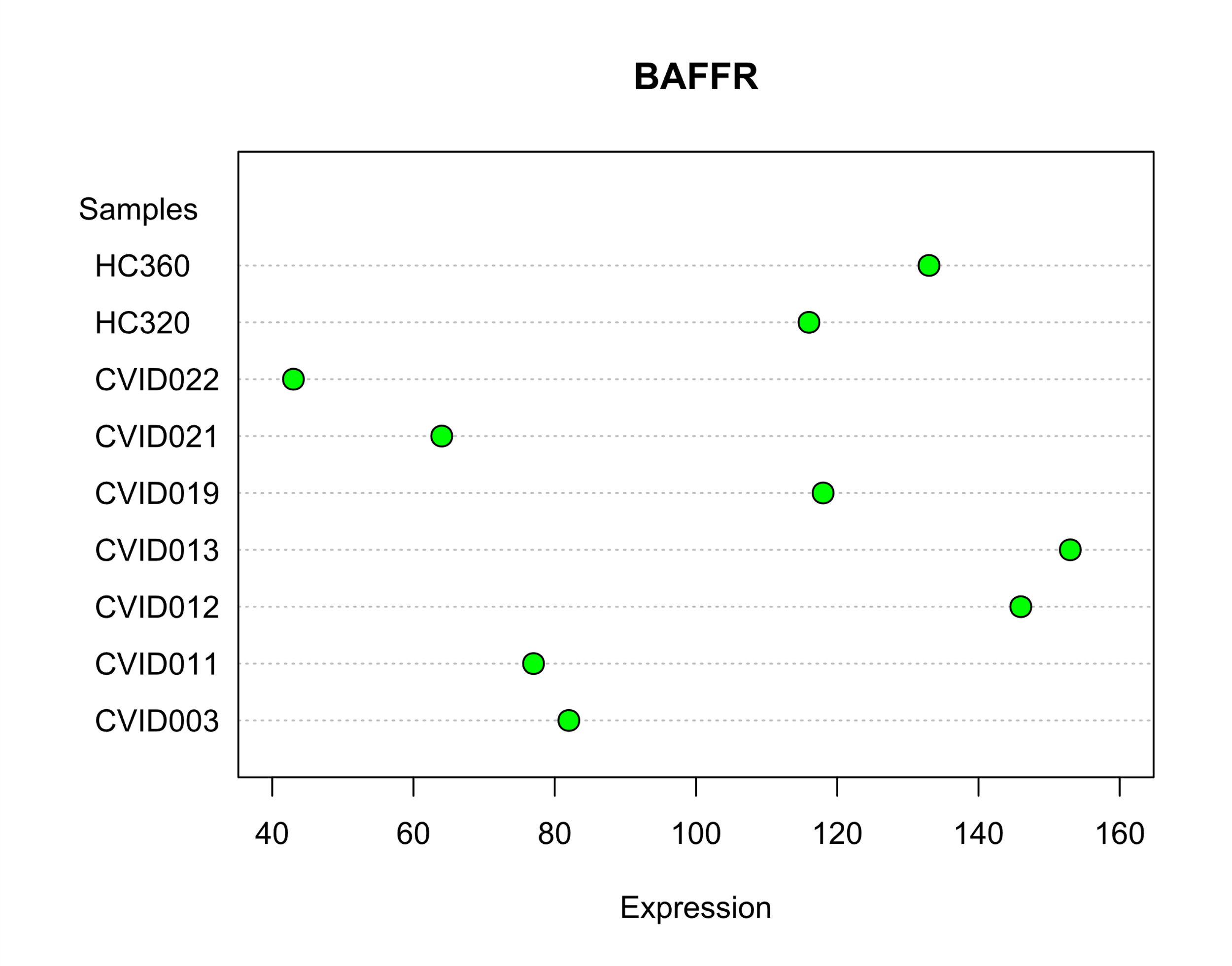


**Supplementary Figure** **3.** BAFFR mRNA expression in Tfh cells from two HCs and seven CVID patients. The index patient is named as CVID013; the dataset is available at https://doi.org/10.1002/eji.202149480


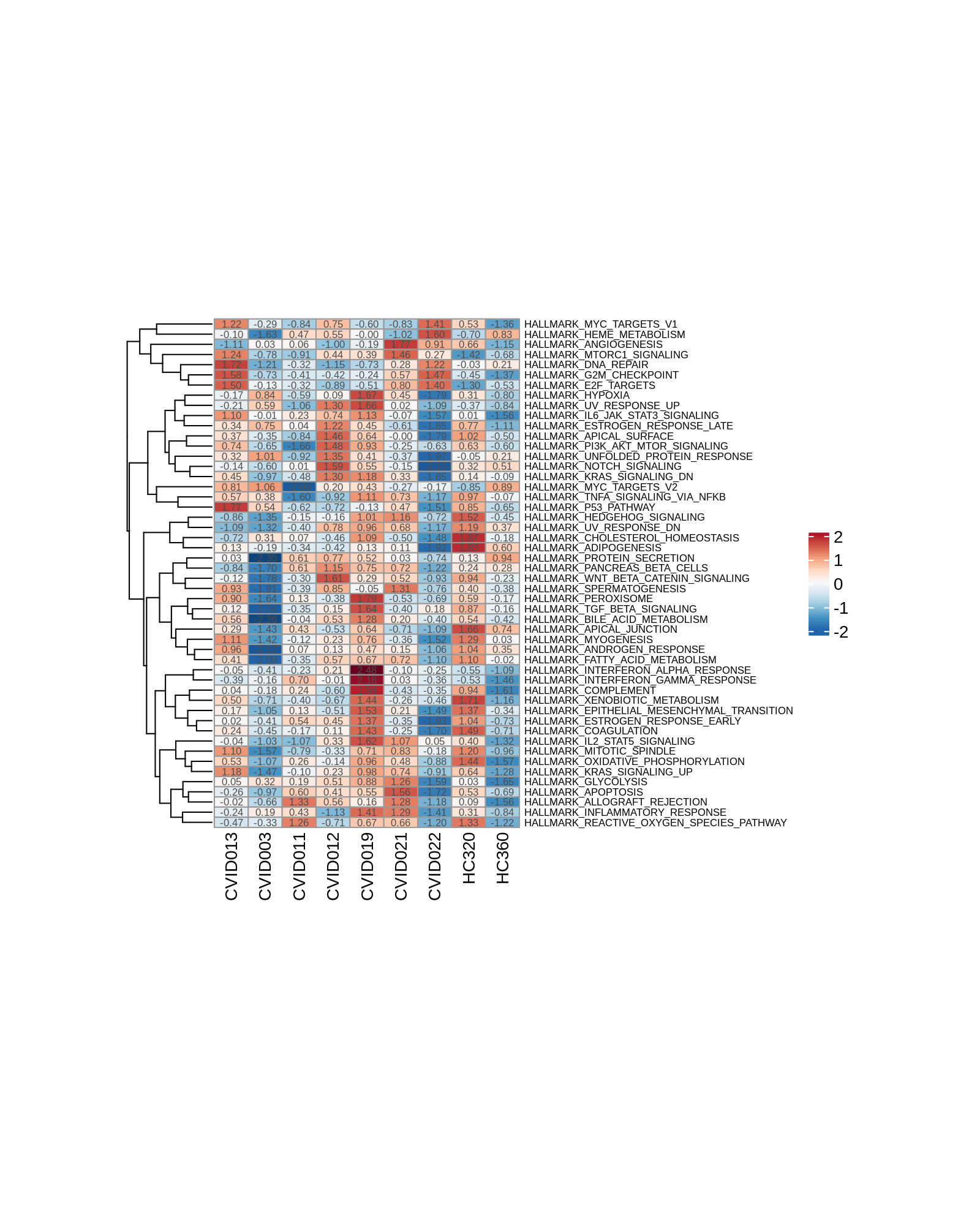


**Supplementary Figure** **4.** Heatmap showing the functional enrichment score, achieved using the ssGSEA approach, for each HALLMARK pathways according to the MiSig Database, in sorted Tfh of the index patient (CVID0013) compared to other age- and sex-matched CVID patients and healthy controls; the dataset is available at https://doi.org/10.1002/eji.202149480

**
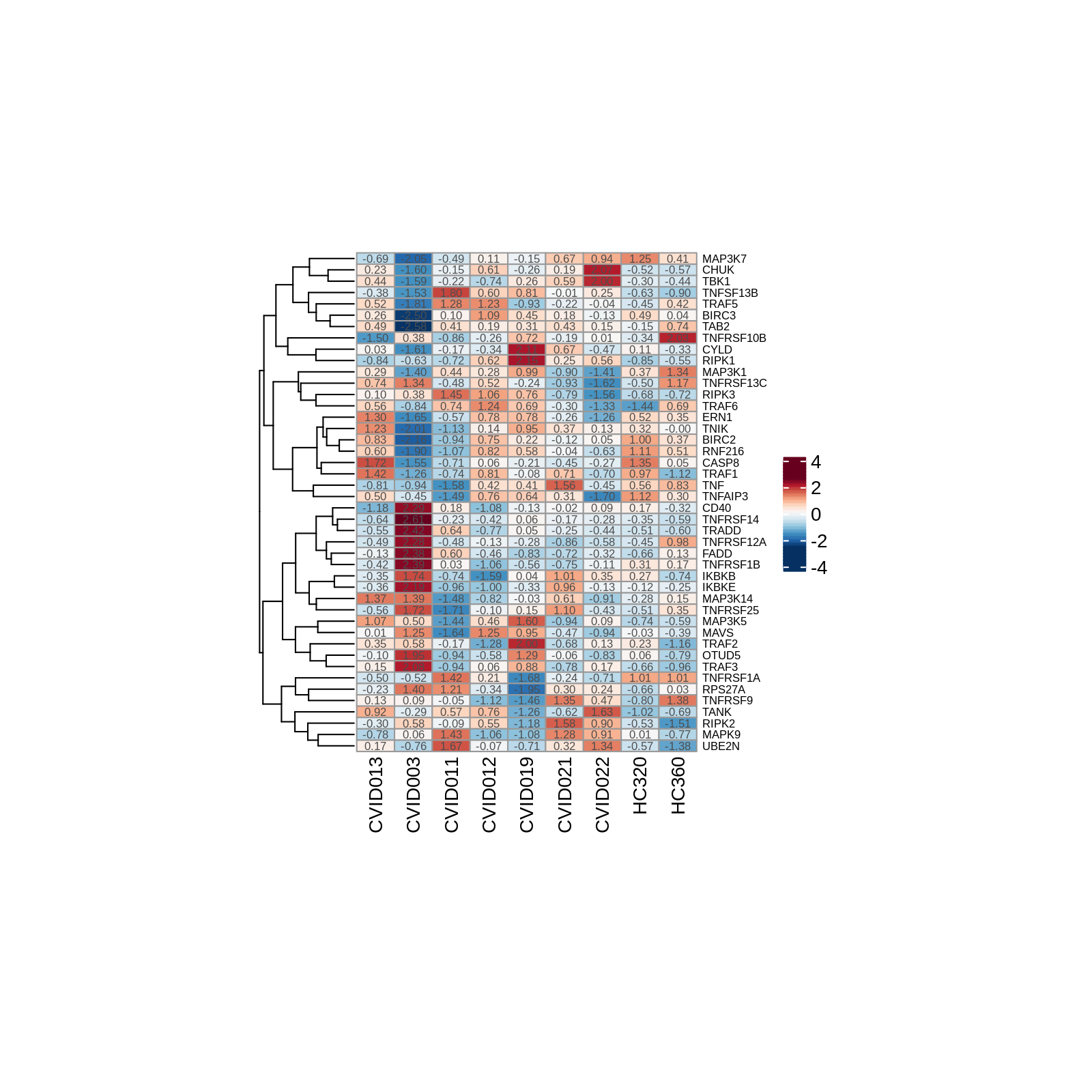
**

**Supplementary Figure** **5.** Heatmap showing the expression of BAFF-R interactors in Tfh cells, according to the STRING Database, in the index patient (CVID0013) compared to other CVID patients and healthy controls; the dataset is available at https://doi.org/10.1002/eji.202149480.


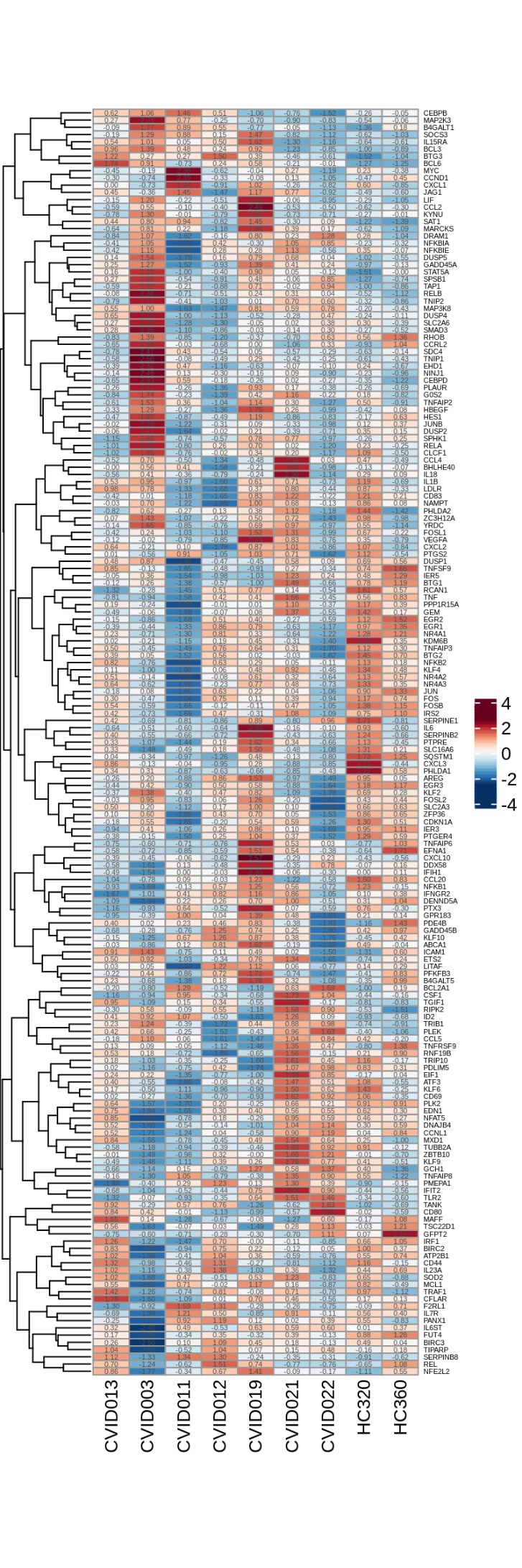


**Supplementary Figure** **6.** Heatmap showing the expression of genes belonging to the TNF signaling via NFKB pathway in Tfh cells according to the MiSig Database in the index patient (CVID0013) compared to other CVID patients and healthy controls; the dataset is available at https://doi.org/10.1002/eji.202149480.

**
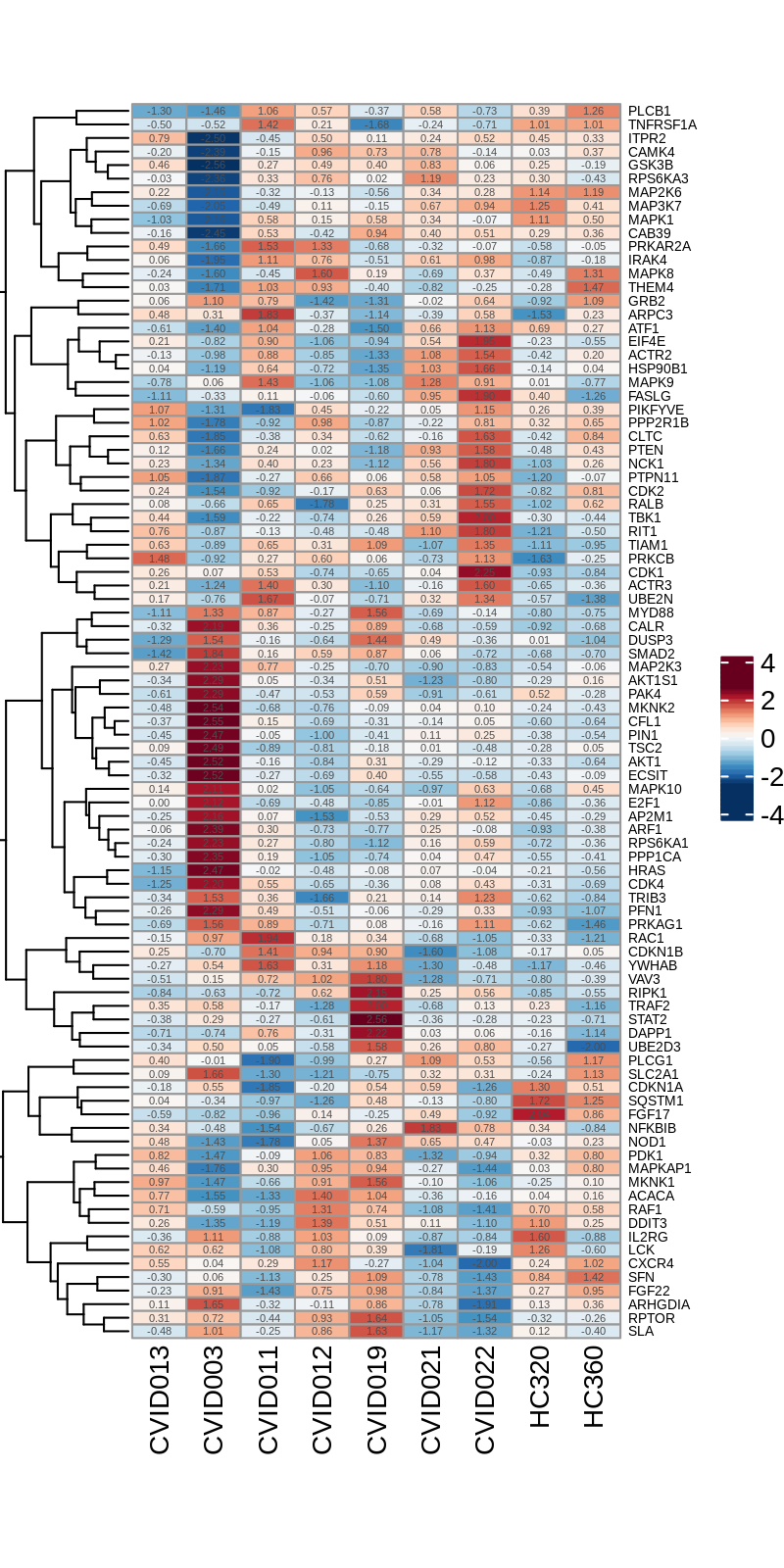
**

**Supplementary Figure** **7.** Heatmap showing the expression genes belonging to the PI3K/AKT/MTOR signaling pathway in Tfh cells according to the MiSig Database, in the index patient (CVID0013) compared to other CVID patients and healthy controls; the dataset is available at https://doi.org/10.1002/eji.202149480.
